# Cell‐Free DNA Blood Collection Tubes Are Appropriate for Clinical Proteomics: A Demonstration in Colorectal Cancer

**DOI:** 10.1002/prca.201700121

**Published:** 2018-03-30

**Authors:** Juhura G. Almazi, Peter Pockney, Craig Gedye, Nathan D. Smith, Hubert Hondermarck, Nicole M. Verrills, Matthew D. Dun

**Affiliations:** ^1^ Priority Research Centre for Cancer Research Innovation and Translation Hunter Medical Research Institute Cancer Research Program School of Biomedical Sciences and Pharmacy Faculty of Health and Medicine University of Newcastle Callaghan NSW Australia; ^2^ School of Medicine and Public Health Faculty of Health and Medicine University of Newcastle Callaghan NSW Australia; ^3^ Analytical and Biomolecular Research Facility Advanced Mass Spectrometry Unit University of Newcastle Callaghan NSW Australia

**Keywords:** Blood collection tubes, Colorectal cancer, Discovery and targeted proteomics, Plasma protein biomarkers

## Abstract

**Background:**

Optimized blood collection tubes (BCT) have been developed to expand the utility of plasma cell‐free DNA (cfDNA) and are in clinical use. The appropriateness of plasma collected and stored in these tubes for proteomic analysis is unknown.

**Methods:**

Paired blood samples were collected in BCT and traditional K3EDTA (EDTA) tubes from healthy controls and from colorectal cancer (CRC) patients before and after surgery, and stored for between 45 min and 48 h at room temperature. Plasma proteins were analyzed following high‐abundant plasma protein depletion in quantitative discovery and targeted proteomics by liquid chromatography tandem‐mass spectrometry (LC‐MS/MS).

**Results:**

BCT reduced cellular protein contamination in healthy controls over time, and increased the number of high confident low‐abundant protein identifications in CRC blood samples compared to matched samples collected in EDTA tubes. The known CRC plasma protein biomarker, carcinoembryonic antigen (CEA), showed elevated levels across patients pre‐operatively when collected and stored in BCT compared to EDTA tubes. Emerging CRC biomarkers, Dickkopf‐3 (DKK3) and Gelsolin (GSN), showed elevated levels pre‐operatively when collected in BCT.

**Conclusions:**

Optimized BCT are appropriate for low‐abundant plasma protein analysis and can be used with confidence for clinical proteomics.

Simultaneous analyses of blood samples at the genomic, transcriptomic, and proteomic levels are increasingly being performed, particularly in the context of proteogenomics.^1^, ^2^ Therefore, determining the optimal conditions for collection and storage of blood samples is essential for future investigations. Assessment of the genetic basis of cancer using biomarkers obtained noninvasively holds promise both for improved personalized anticancer treatment and for the screening of asymptomatic individuals. Common cancers such as colorectal cancer (CRC) are curable with early detection, therefore biomarkers such as circulating cell‐free tumor DNA (cfDNA) and tumor‐associated plasma proteins are valuable tools for the effective management of CRC.^3^, ^4^

**Clinical Relevance**

Plasma cell‐free DNA (cfDNA) is emerging as a clinically relevant biomarker for the management and surveillance of cancer. Optimized blood collection tubes (BCT) have been developed to prolong the stability and integrity of cfDNA over time, but the utility of plasma proteins stored in these tubes for subsequent proteomic analysis is unknown. In this discovery and targeted quantitative proteomic study, we demonstrate for the first time that plasma stored for 48 h at room temperature in optimized BCT showed improved capacity to detect known low‐abundance CRC protein biomarkers such as carcinoembryonic antigen (CEA) when compared to standard EDTA collection tubes in matched samples. This unexpected finding increases the opportunity for plasma protein biomarker research and detection, and may allow for the simultaneous detection of DNA and protein in blood. This has implications for standardized blood collection protocols in clinical laboratories for the noninvasive detection of plasma‐based biomarkers

Carcinoembryonic antigen (CEA) is a plasma protein biomarker used in disease progression for metastatic CRC^5^; however, the detection of CEA has not proven to be sufficiently sensitive for detecting primary early stage CRC.^6^ cfDNA is detected in almost all patients with solid tumors including CRC,^7^ with levels paralleling that of CEA. cfDNA can be detected in CRC patients with as few as 50 million cancer cells.^5^ However, in the clinical setting, the efficiency of processing of patients’ blood samples is highly variable, which has deleterious effects on the quality and long‐term utility of both cfDNA and protein. Cellular nucleic acid and protein contamination as a result of lysis of nucleated and non‐nucleated cells is common,^8^ therefore specialized blood collection tubes (e.g., PAXgene or Streck; referred to as “BCT”) have been developed to reduce plasma DNA (pDNA) contamination. BCT enhance low‐abundance DNA target detection to allow for accurate cfDNA quantification aiding in the molecular characterization of cancer.^9^ Additionally, circulating tumor cells (CTC) are reported to be stored with higher stability in BCT compared to EDTA tubes.^10^ CTCs are useful for the detection of advanced stage CRC.^11^ Importantly, both cell‐free and cell‐based cancer biomarkers provide specific genetic information on primary and metastatic cancers and their stability is therefore an important consideration during the establishment of plasma biobanks.

Processing time, proteolytic degradation, and changes to protein structure are important considerations for the maintenance of high‐quality clinical proteomic biospecimens for biomarker discovery, verification, and validation.^9^ No data is available on the quality and stability of proteins stored in BCT. Given that the majority of plasma biomarkers used for cancer detection and diagnosis are proteins, we sought to determine whether plasma stored in BCT tubes was amenable to downstream proteomic characterization.

Matched blood samples were collected from three healthy controls in both BCT and EDTA tubes, and stored for 1, 6, 24, and 48 h prior to plasma processing. Using established methods including high‐abundant protein depletion (Figure 1, Supporting Information)^12^ and tryptic peptide preparation,^13^ each sample was subjected to discovery label‐free data‐dependent acquisition (DDA) LC‐MS/MS (Figure 1A) (detailed materials and methods available in Supporting Information). No difference in the number of high confident (two unique peptides per protein using a FDR 1%) protein identifications were observed in healthy control samples processed 1 h after collection in either BCT or EDTA tubes, highlighting the appropriateness of plasma for proteomics when stored in BCT (Figure 1B). After 6 h of storage at room temperature prior to processing, an overall increase in total protein identifications was observed, suggesting an increase in contamination of cellular proteins over time. This was significantly mitigated when blood samples were collected and stored in BCT (Figure 1B). Analysis of the potential source of contamination identified intracellular proteins such as the molecular chaperone heat shock 70 kDa protein 5 (HSPA5), signaling proteins such as Hedgehog‐interacting protein (HHIP) and regulatory‐associated protein of mTOR, RAPTOR (RPTOR), and nuclear protein nuclear distribution protein nudE‐like 1 (NDEL1) in EDTA tubes (found in each of the EDTA health controls), whereas new proteins identified in BCT were uniquely secretory proteins, like the multidrug resistance protein 1 (ABCB1) and collagen alpha‐1(I) chain (COL1A1) (found in each of the BCT healthy controls). The significant increase in cellular protein contamination continued in healthy controls collected in EDTA tubes at each of the additional time points. Overall, blood samples stored in EDTA tubes were significantly more susceptible to cellular protein contamination, potentially as a result of cell lysis, compared to BCT (*p‐*value < 0.0001) (Figure 1B). This may contribute to masking of low‐abundant plasma proteins, and have important clinical implications for the detection of low‐abundant known and novel plasma biomarkers by mass spectrometry.

**Figure 1:**
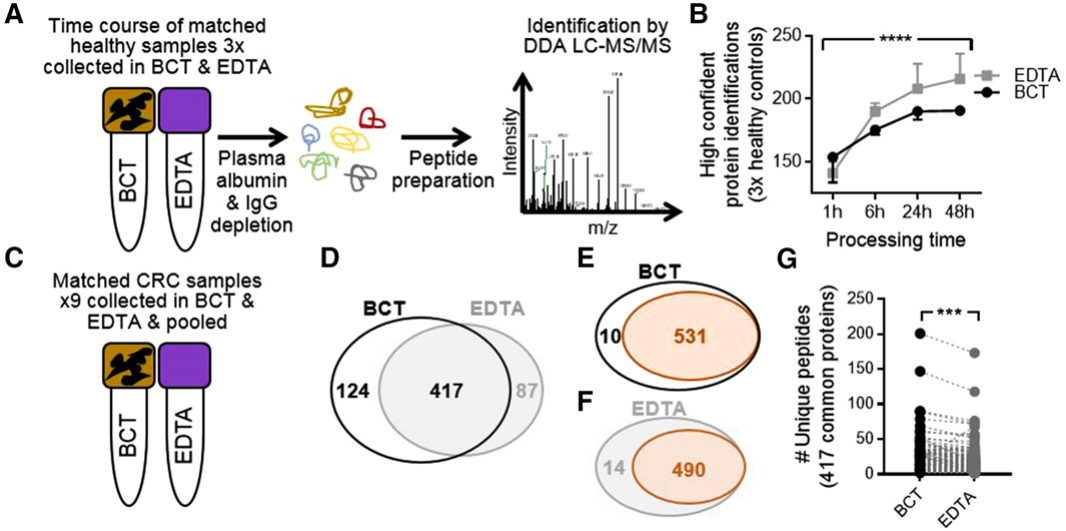
Suitability of plasma proteins for proteomics when collected and stored in optimized BCTs. A) Workflow for the time course of paired healthy control blood samples collected in both BCT and EDTA tubes prior to LC‐MS/MS via DDA. B) Number of high confidence protein identifications (two unique peptides per proteins, FDR 1%) following storage of healthy controls blood samples for 1, 6, 24, and 48 h at room temperature in BCT and EDTA tubes prior to processing (****p‐value < 0.0001, two‐way ANOVA). C) Workflow for CRC patient blood samples collection and pooling. D) Venn diagram of CRC using two unique peptides per protein in both tube types. Venn diagram of proteins mapping to the Plasma Protein Database (orange) from pooled samples collected in E) BCTs or F) EDTA tubes prior to processing. G) Significantly increased numbers of unique peptides per protein were identified when collected and stored in BCT, compared to EDTA tubes (*p‐*value < 0.0001, paired *t*‐test).

To assess whether blood samples from CRC patients (Table 1, Supporting Information) collected in BCT and EDTA tubes were amenable to proteomics via DDA analysis, samples were pooled from nine CRC patients in matched BCT and EDTA tube collections (Figure 1C) and processed as in Figure 1A. These samples were obtained from known CRC patients prior to endoscopy and resection, and processed following 45 min–24 h of storage. Using a reduced stringency reporting criteria (i.e., one unique peptide per protein, however FDR 1%), we identified 1063 proteins in pooled BCT collections and 1119 in matched EDTA collections. However, when we employed a stringent reporting criterial (minimum of two unique peptides per protein, FDR 1%), DDA analysis identified 541 unique proteins from blood samples collected and stored in BCT, and 504 unique proteins in paired samples collected in EDTA tubes. A total of 417 proteins were common to both tubes (Figure 1D and Table 2A, Supporting Information). The Plasma Proteome Database^14^ was used to interrogate proteins identified, revealing 98.2% and 97.2% of proteins identified following storage in BCTs or EDTA tubes, respectively, were from plasma (Figure 1E,F). Comparative analysis of unique peptides per protein for the 417 proteins identified to be common to both tube types revealed a significant increase (*p‐*value < 0.0001) in the number of unique peptides sequenced per protein (average per protein; BCT = 11, EDTA = 9, total number of unique peptides; BCT = 4568 and EDTA = 3717) when blood samples were collected and stored in BCT (Figure 1G and Table 2B, Supporting Information). This highlights the utility of BCT for CRC plasma sample proteomic analysis, suggesting that the increased maintenance of cellular integrity afforded by the proprietary cell stabilization components of BCT affords deeper sequence coverage of low‐abundant proteins that comprise the plasma proteome.

These qualitative studies revealed a difference in the number of unique peptides identified per protein between collection tube types; however, to further assess the utility of plasma proteins to be used in comparative and quantitative proteomic analysis, tandem‐mass tagging (TMT) of peptides, coupled with offline hydrophilic interaction chromatography (HILIC),^15^ from matched patient samples collected in BCT and EDTA tubes was performed (2 × TMT ten‐plex)^16^ (Figure 2A and Figure3B and Table 3, Supporting Information). Correlation of the relative abundance of matched peptides in BCT versus EDTA tubes showed no difference in reporter‐ion abundance (mean, R^2^ = 88.3%, +/−5.4%, SD = 4.8%) when samples were collected and stored at RT for very short periods prior to processing (45–90 min, Figure 2B). However, when processing of samples was performed at clinically achievable time points (i.e., 19–24 h post collection), the correlation between BCT and EDTA tubes was less linear (mean, R^2 = ^71.4%, +/−9.5%, SD = 6.5%) (Figure 2C). Reporter‐ion abundance for many peptides increased in samples prepared from blood samples stored for 19–24 h post collection in BCT prior to peptide tagging and HILIC LC‐MS/MS (Figure 2C, dashed circles).

**Figure 2:**
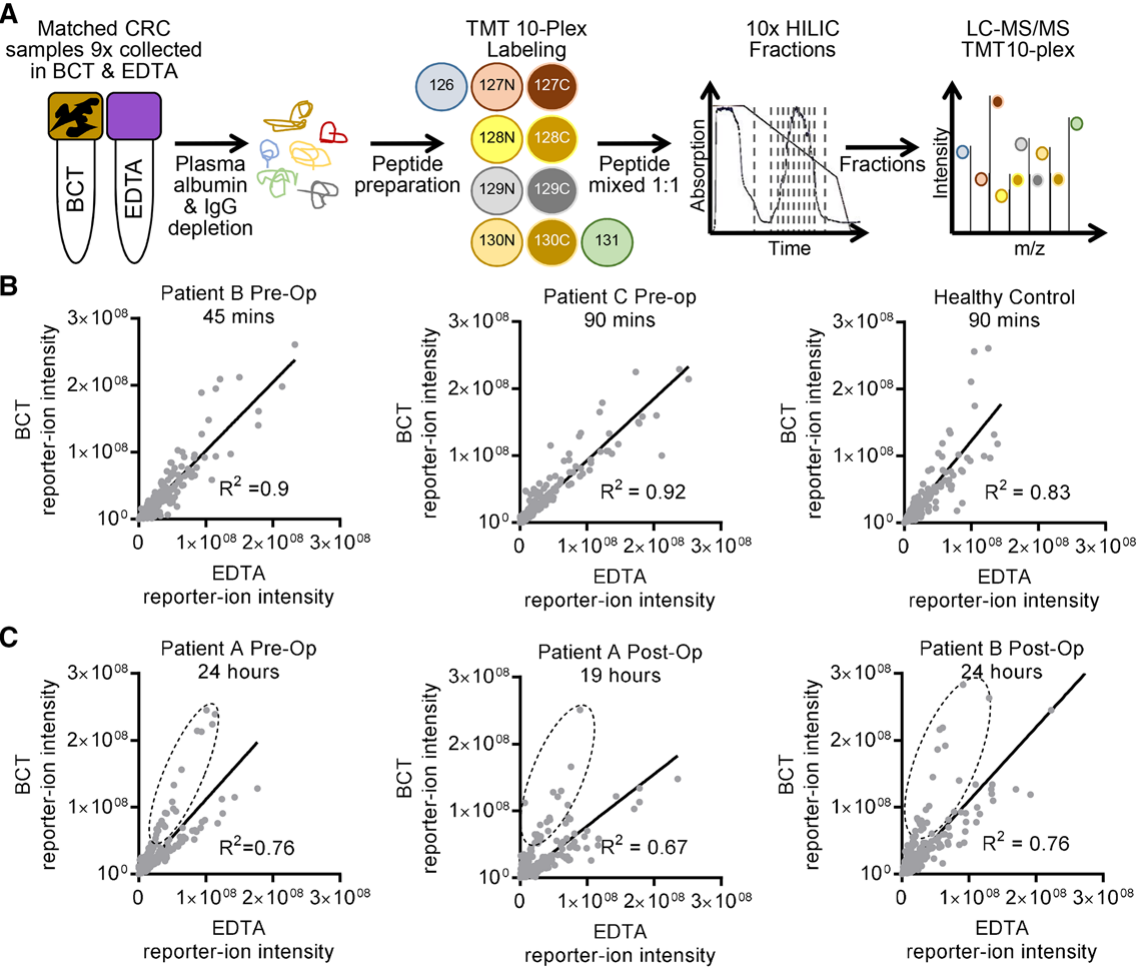
Linear correlation of peptide abundance using TMT from individual patients stored in BCT or EDTA. A) Workflow for matched CRC patient sample collection and processing for comparative and quantitative analysis (TMT) using BCT and EDTA tubes. B) Relative protein abundance using paired patient samples collected in BCT or EDTA and stored for short durations before processing. C) Analysis was repeated using paired samples stored from between 19 and 24 h post collection. Circled area represents paired peptides that show great reporter‐ion abundance in BCT versus EDTA tubes.

Comparative and quantitative analyses identified 641 unique proteins across all samples (Table 4A,B, Supporting Information). Of these, 184 proteins showed +/−0.5 log2 fold change in peptide abundance pre‐ versus post‐operation in BCT and EDTA tubes, (high‐grade adenocarcinoma and low‐grade adenocarcinoma). Of the 184 proteins, 7 showed +/−0.5 log2 fold change across both tube types (Figure 3 and Table 5, Supporting Information). Proteins with higher abundance pre‐operatively compared to post‐operatively included gelsolin (GSN), structural maintenance of chromosomes protein 1B (SMC1B), E3 ubiquitin‐protein ligase SHPRH (SHPRH), and semaphorin‐3C (SEMA3C) (Figure 3, Supporting Information).

To validate these, and to test the well‐characterized CRC plasma protein biomarkers, we used nine paired CRC patient plasma samples collected in both BCT and EDTA, pre‐ and post‐operatively (36 total samples, Table 1, Supporting Information), and performed targeted proteomics using parallel reaction monitoring (PRM)^17^ (Figure 3A).

**Figure 3:**
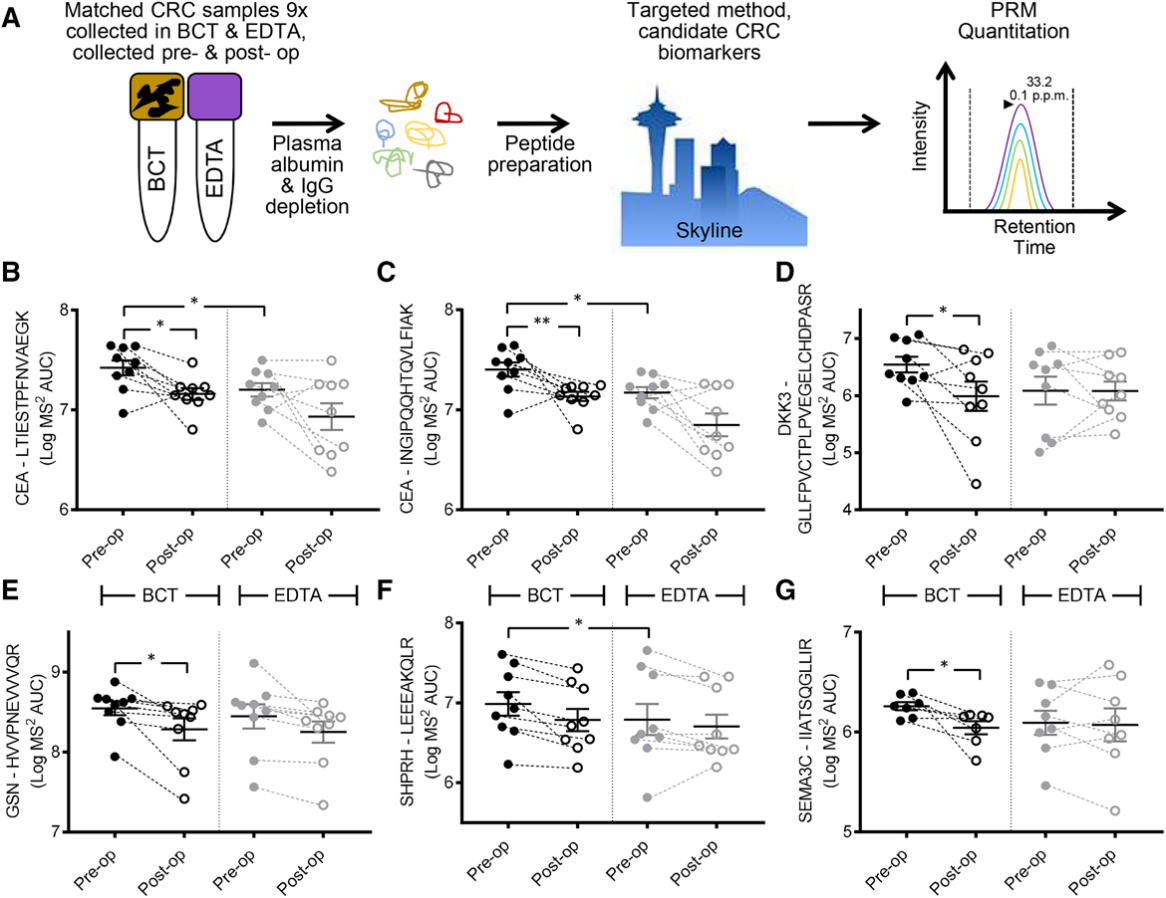
PRM analysis on known and potential novel CRC plasma protein biomarkers. A) Workflow for quantitative validation of CRC biomarkers by targeted proteomics via PRM. PRM was performed on nine CRC patient plasma samples collected in both BCT and EDTA, pre‐ and post‐operatively. A–C) PRM quantification CRC plasma protein biomarker CEA, D) DKK, E) GSN, F) SHPRH, and G) SEMA3C (*p‐value < 0.05, **< 0.01, Mann–Whitney *U* Wallis used to determine statistical differences pre‐ versus post‐operation, Wilcoxon matched‐pairs signed rank tests to determine statistical differences between matched tube type collections pre‐operatively).

CEA is the most well‐characterized CRC blood‐based biomarker.^4^ Using two unique peptides, and the log transformed AUC for matched *y‐* and *b‐*ion transitions (minimum of four with MS^2^ accuracy <10 ppm), we used Mann–Whitney *U* Wallis nonparametric testing to determine statistical differences between CEA pre‐ versus post‐operation. CEA was more abundant pre‐operatively when collected in BCT compared to EDTA tubes (peptide #1 Figure 3B, *p‐*value = 0.0173; peptide #2 Figure 3C, *p‐*value = 0.0098). PRM analysis of CEA in samples collected in EDTA tubes for both peptides showed a nonsignificant trend for decreased abundance post‐operatively. Interestingly, comparing the abundance of both peptides for CEA between BCT and EDTA tubes pre‐operatively showed increased absolute abundance of CEA peptides when collected in BCTs (#1 and #2 *p‐*value = 0.0195, Wilcoxon matched‐pairs signed rank tests). As each unique peptide for CEA showed significantly higher abundance pre‐operatively when collected in EDTA tubes, the choice of collection tube type could have important research and clinical implications when using CEA for the detection of CRC via mass spectrometry.

DKK3 has been shown to be a useful biomarker when used in a protein biomarker panel with IGFBP2 and PKM2, and shown to have the equivalent sensitivity to the fecal occult blood test (FOBT) for the detection of early stage CRC.^18^ Given that the majority of our patients were diagnosed with low‐grade adenocarcinoma following endoscopy (8× Stage I–IIA and 1× Stage IIIB), we examined these three biomarkers by PRM (all three were detected during our comparative and quantitative analyses, Table 4, Supporting Information). Peptides mapping to these proteins were then quantified by PRM. Only DKK3 showed higher abundance in BCT compared to EDTA tubes pre‐operatively (*p‐*value = 0.0278, Wilcoxon matched‐pairs signed rank tests) (Figure 3D).

GSN has been shown to be increased in the plasma of advanced stage CRC patients by ELISA^19^ and also by isobaric tags–based quantitative LC‐MS/MS^20^ (Figure 3C and Table 5, Supporting Information). GSN significantly decreased post‐operatively when collected in BCT (BCT *p‐*value = 0.04), with no difference in total abundance between tube types pre‐operatively (Figure 3E) when measured by PRM. The total abundance of SHPRH was higher in BCT versus EDTA tubes pre‐operatively (*p‐*value = 0.0273) (Figure 3F), whereas SEMA3C was significantly higher pre‐ versus post‐operatively only when collected in BCT (SEMA3C *p‐*value = 0.0379) (Figure 3G).

The results of this study show for the first time the suitability of human plasma proteins for qualitative and quantitative proteomic analysis after collection and storage for up to 48 h at room temperature in cfDNA‐optimized BCT. These tubes overcome some of the issues that delays in processing time, temperature, and handling contribute to the deterioration of non‐protein–based biomarkers. Numerous studies now report that optimized BCT maintain the integrity of cfDNA^21^ and CTC,^22^ and now the utility of plasma proteins for proteomics is herein established. It is important to note that all CRC patients used in this study had confirmed cases of CRC, and were not asymptomatic, and that processing time varied from 45 min to 24 h (48 h for healthy controls). Future, further expanded studies are necessary to test the clinical relevance of these proteins for diagnosis, screening, and staging. Future research is likely to show that a combinatorial panel of CRC protein and non‐protein biomarkers, such as cfDNA, will provide us with the most accurate indication of disease, particularly in the asymptomatic setting. In the age of proteogenomics, performing simultaneous discovery of protein and non‐protein biomarkers using samples collected and stored in the same optimized tubes holds promise for improved early detection and increased cures for cancers such as CRC.

**Abbreviations**
BCT: blood collection tubes
CEA: carcinoembryonic antigen
cfDNA: circulating cell‐free tumor deoxyribonucleic acid
CRC: colorectal cancer
CTC: circulating tumor cells
DDA: data‐dependent acquisition
FOBT: fecal occult blood testing
HILIC: hydrophilic interaction liquid chromatography
TMT: tandem mass tag

## Conflict of Interest

The authors declare no conflict of interest.

## Supporting information

Supporting information.

Supporting information.

Supporting information.
