# Supplementary material for: Cell‐Free DNA Blood Collection Tubes Are Appropriate for Clinical Proteomics: A Demonstration in Colorectal Cancer
Source: Proteomics Clin Appl. 2018 Mar 30;12(3):1700121. doi: 10.1002/prca.201700121 (PMC5947838; doi:10.1002/prca.201700121)
Supplement: Supplementary file 1 — Supporting information. [file PRCA-12-na-s001.pptx]

## Slide 1
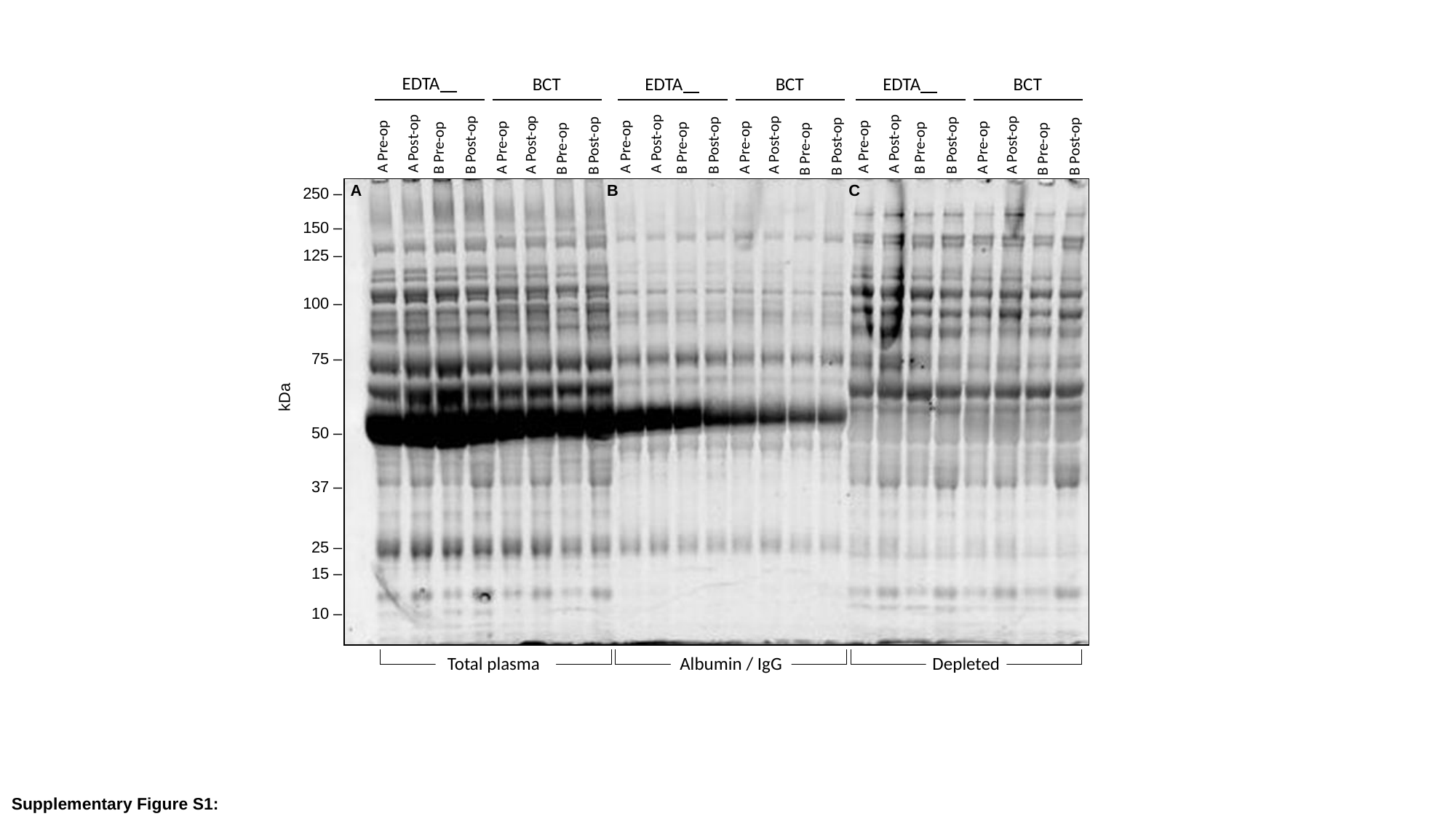

EDTA
BCT
EDTA
EDTA
BCT
BCT
A Pre-op
A Pre-op
A Pre-op
B Post-op
A Post-op
B Post-op
B Post-op
A Post-op
A Post-op
B Post-op
B Post-op
B Post-op
A Post-op
A Pre-op
A Post-op
A Post-op
A Pre-op
A Pre-op
B Pre-op
B Pre-op
B Pre-op
B Pre-op
B Pre-op
B Pre-op
Depleted
Total plasma
Albumin / IgG
A
B
C
250 –
150 –
125 –
100 –
75 –
kDa
50 –
37 –
25 –
15 –
10 –
Supplementary Figure S1:

## Slide 2
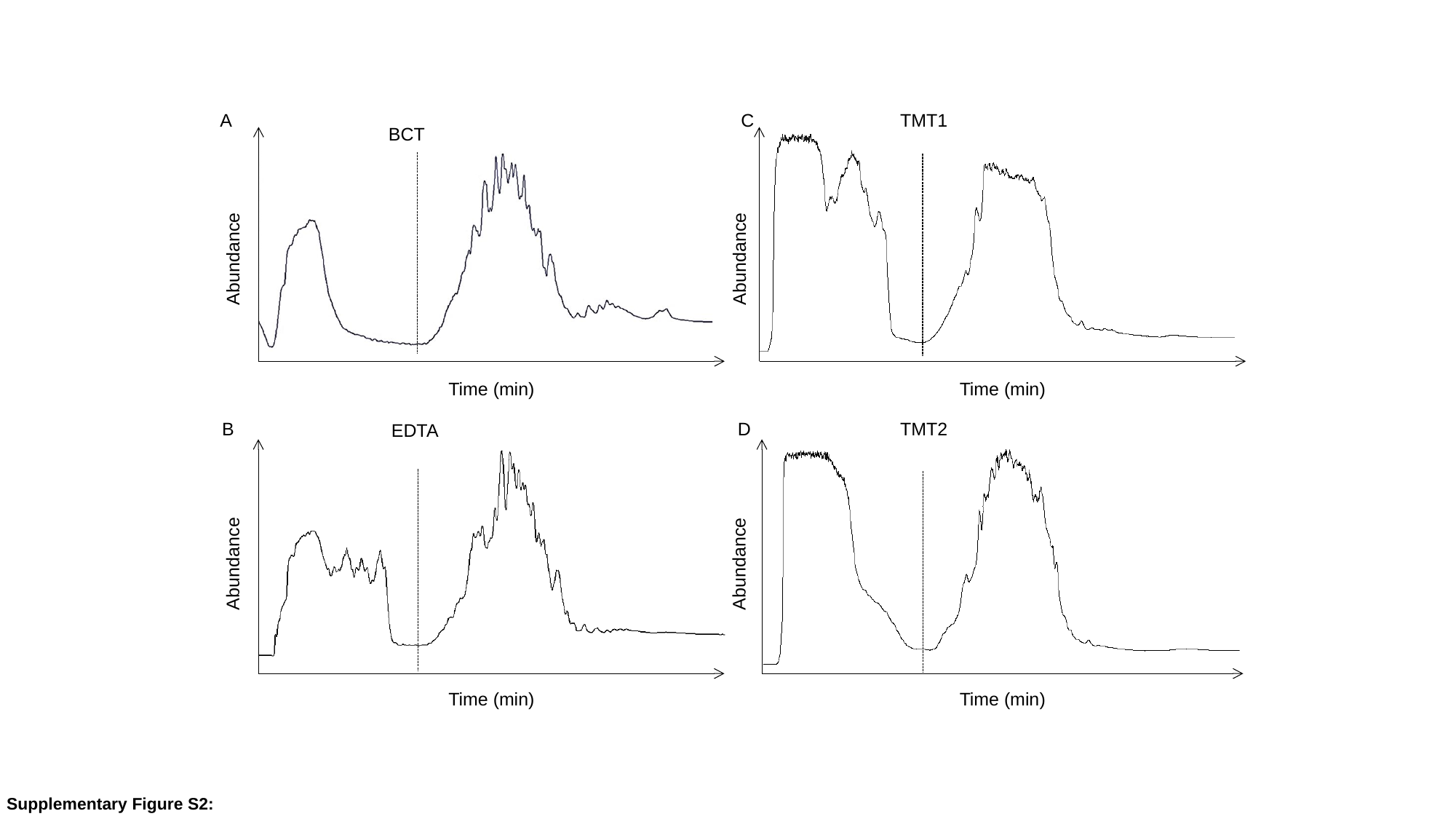

A
C
TMT1
BCT
Abundance
Abundance
Abundance
Abundance
Time (min)
Time (min)
B
D
TMT2
EDTA
Time (min)
Time (min)
Supplementary Figure S2:

## Slide 3
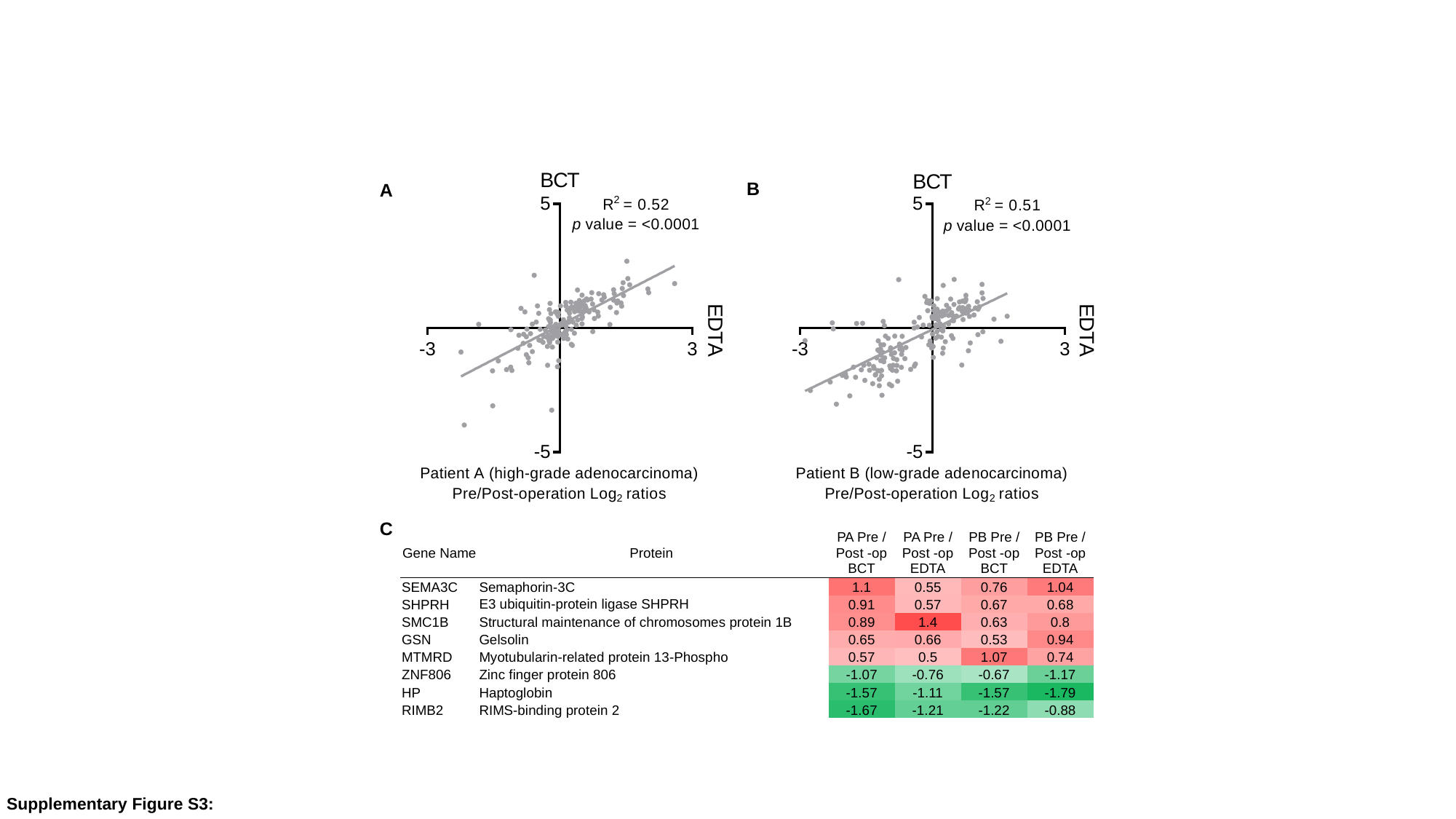

B
A
C
| Gene Name | Protein | PA Pre / Post -op BCT | PA Pre / Post -op EDTA | PB Pre / Post -op BCT | PB Pre / Post -op EDTA |
| --- | --- | --- | --- | --- | --- |
| SEMA3C | Semaphorin-3C | 1.1 | 0.55 | 0.76 | 1.04 |
| SHPRH | E3 ubiquitin-protein ligase SHPRH | 0.91 | 0.57 | 0.67 | 0.68 |
| SMC1B | Structural maintenance of chromosomes protein 1B | 0.89 | 1.4 | 0.63 | 0.8 |
| GSN | Gelsolin | 0.65 | 0.66 | 0.53 | 0.94 |
| MTMRD | Myotubularin-related protein 13-Phospho | 0.57 | 0.5 | 1.07 | 0.74 |
| ZNF806 | Zinc finger protein 806 | -1.07 | -0.76 | -0.67 | -1.17 |
| HP | Haptoglobin | -1.57 | -1.11 | -1.57 | -1.79 |
| RIMB2 | RIMS-binding protein 2 | -1.67 | -1.21 | -1.22 | -0.88 |
Supplementary Figure S3:
