# Supplementary material for: Cell‐Free DNA Blood Collection Tubes Are Appropriate for Clinical Proteomics: A Demonstration in Colorectal Cancer
Source: Proteomics Clin Appl. 2018 Mar 30;12(3):1700121. doi: 10.1002/prca.201700121 (PMC5947838; doi:10.1002/prca.201700121)
Supplement: Supplementary file 2 — Supporting information. [file PRCA-12-na-s002.docx]

**SUPPLEMENTARY INFORMATION**

**SUPPLEMENTARY MATERIALS AND** **METHODS**

**Reagents**

The following reagents were used: cfDNA and CTC optimized blood collection tubes (BCT) (Streck® St La Vista, NE, USA), K3EDTA blood collection tubes (Greiner, Kremsmünster, Austria), Complete mini EDTA-free tablet Protease inhibitor cocktail (PIC) (Roche, Basel, Switzerland). ProteoPrep Blue Albumin and IgG Depletion Kit, Triethylammonium bicarbonate (TEAB), Thiourea, DL-Dithiothreitol (DTT) and iodoacetamide (IAA) (Sigma-Aldrich, Saint Louis, MO, USA). 4-12% gradient Bis-Tris NuPAGE precast gels, SYPRO® Ruby Protein Gel Stain, BCA assay kit, TMT10plex™ Isobaric Labelling 0.8 mg kit, Qubit protein assay kit; (Thermo Fisher Scientific, MA, USA). Precision Plus Protein™ Kaleidoscope™ Prestained Protein Standards (Bio-Rad, NSW, Australia). Urea, Thiourea (VWR, QLD, Australia) and Trp/Lys-C enzyme mix (Promega, Madison, WI, USA). Poros Oligo R3 reversed phase material (PerSeptive Biosystems, Framingham, MA, USA). C18 Empore disk plug (3M, St. Paul, MN, USA).

**Patient Samples**

Blood from 3 healthy controls and 9 CRC patients (median age of 67 +/- 12 years) (Supplementary Table S1) were collected in both in BCT and EDTA tubes, on the day of operation (pre-op) and 1-2 months after the operation (post-op). Additionally, blood samples were collected from healthy volunteers in both types of collection tubes (40 samples in total). The time from phlebotomy collection to processing was recorded for all samples but varied from 45 minutes to 24 hours, in keeping with typical clinical workflows. Each clinical sample was collected as part of the sequential blood collection feasibility study of the Hunter Cancer Biobank (HNEHREC Ref No. 14/12/10/4.02). Healthy volunteers were recruited under institutional ethics approval. Samples were centrifuged following storage (according to Table 1) for 15 minutes at 1600 RPM. Plasma was collected, separated into 1 ml aliquots and stored at -80°C. Samples to be analyzed by proteomics were thawed on ice.

**Plasma Albumin Depletion**

The ProteoPrep Blue Albumin and IgG Depletion Kit was used to deplete the most abundant proteins prior to mass spectrometry, in accordance with the manufacturer’s instructions. Briefly, 25 µL of plasma was loaded onto the equilibrated column and incubated two consecutive times and the "twice depleted" plasma was collected (depleted fraction). The bound proteins (Albumin / IgG) were then eluted using Protein Extraction Reagent.

**Protein quantitation and SDS-PAGE**

The concentration of proteins in the depleted and bound fractions was quantified using a bicinchoninic acid (BCA) assay. 15 µg of proteins were resolved in reducing conditions on 4-12% gradient Bis-Tris NuPAGE precast gels [28]. Following electrophoresis, gels were stained with SYPRO® Ruby Protein Gel Stain, according to manufacturer’s instructions, and visualized on a Fuji LAS4000 [29].

**Protein Digestion and Purification**

100 µg of albumin/IgG-depleted plasma proteins were denatured using 6M urea, 2M thiourea, and reduced using 10 mM DTT for 30 minutes at 55oC. Reduced proteins were alkylated using 20 mM IAA for 30 minutes at 55oC in the dark. Denatured proteins were then digested using 1:50 ratio of Trp/Lys-C to protein for 3 hours at room temperature (RT). The concentration of urea was then reduced below 1M by adding 4 volumes of 20 mM TEAB pH 7.8 overnight at 37 °C. Peptides were quantitated using Qubit protein assay kit [13].

**Tandem mass tag (TMT) 10-Plex Peptide Labeling**

To perform unbiased global quantitative proteomic profiling of CRC patients pre and post-operation tandem mass tag (TMT) was performed [30]. Peptides were labeled and mixed according to the manufacturers' instructions (see labeling strategy in Supplementary Table S3). Following labeling and mixing, peptides were purified using OligoR3-C18 modified StageTip microcolumns [31]. Labelling efficiency was determined by LC-MS/MS (described below). The quantity of each labeled peptide set was adjusted 1:1 then checked again by LC-MS/MS. 20 µg of 1:1 TMT-labeled peptides were subjected to hydrophilic interaction chromatography (HILIC) [32] and fractionated into 12 fractions [33].

**Liquid Chromatography Tandem Mass spectrometry (LC-MS/MS)**

Mass spectrometry was performed using a Q-Exactive Plus hybrid quadrupole-Orbitrap MS system (Thermo Fisher Scientific, Bremen, DE) coupled to a Dionex Ultimate 3000RSLC nanoflow HPLC system (as described [14]). Samples were loaded onto an Acclaim PepMap100 C18 75 μm x 20 mm trap column (Thermo Fisher Scientific) for pre-concentration and online desalting. Separation was then achieved over an EASY-Spray PepMap C18 75 μm x 250 mm column (Thermo Fisher Scientific, Bremen, DE), employing a linear gradient from 2% to 32% acetonitrile at 300nl/min over 120 min.

Data Dependent Acquisition (DDA), was performed on the Q-Exactive Plus MS System was operated in full MS/data-dependent MS/MS mode (as described [14]). A precursor ion of endogenous peptide was measured in the Orbitrap scanning the mass range from m/z 390-1400 with an Orbitrap resolution of 70,000, a target automatic gain control (AGC) value of 1e6, and maximum fill times of 50ms. The 20 most intense multiply charged precursors were selected for higher-energy collision dissociation (HCD) fragmentation with a normalized collisional energy (NCE) of 30, the MS/MS fragments were measured at an Orbitrap resolution of 17,500 for unlabeled samples and 35,000 for TMT labeled samples with AGC of 5e5, and maximum fill times of 110ms.

Parallel Reaction Monitoring (PRM) was performed on the Q-Exactive Plus MS System was operated in full MS/data-dependent MS/MS mode (as described [14]) using an inclusion list of targets for peptide. The acquisition methods combined two alternating scan events corresponding to a full scan event and a PRM event targeting the precursor ions selected for endogenous peptides without scheduling. In full scan mode, the Orbitrap scanned the mass range from m/z 390-1500 with a resolution of 17,500, AGC value of 1e6, and maximum fill times of 50ms. Whilst in PRM mode, precursor ions were selected from an inclusion list of targets for peptide fragmentation via HCD at a NCE of 28, then measured with an Orbitrap resolution of 17500, AGC target of 2e5, and maximum fill times of 90 ms.

**Data Analysis**

**Data Dependent Acquisition (DDA):** Database searching of all .raw files was performed using Proteome Discoverer 2.0 (Thermo Fisher Scientific, Bremen, DE). Mascot 2.2.3 and SEQUEST HT were used to search against the Swiss_Prot, Uniprot_human, 152206 sequences, Uniprot_human_proteome_appended_cancer_mutations databases (uniprot_sprot.fasta, downloaded 07/29/2015, 1917646 sequences, uniprot_human_proteome_appended_cancer_mut.fasta downloaded 04/19/2016). Database searching parameters as described [14] using up to 2 missed cleavages to allow for full tryptic digestion, precursor mass tolerance was set to 10 ppm and fragment mass tolerance 0.02 Da with dynamic modifications including oxidation (M), phospho (S/T), phospho (Y) and TMT6plex. Static carbamidomethyl (C). Peptides identified were applied to Percolator on the basis of q-values, which were estimated from the target-decoy search approach. To filter out target peptide spectrum matches (target-PSMs) over the decoy-PSMs, a fixed false discovery rate (FDR) of 1% was set at the peptide level.

**Parallel Reaction Monitoring (PRM):** Signature unlabeled tryptic peptide m/z values were predicted using protein sequences from the NCBI database with the Skyline software 2.0 (MacCoss Lab, University of Washington) [34]. Methods optimized for collision energy, charge state and retention times were generated experimentally (see inclusion list Supplementary Table S6). Targeted MS2 spectra were acquired using a PRM approach [16]. PRM analysis was performed using an inclusion list of proteins identified by our discovery proteomics and performed in duplicate (72 PRM assays). Following this, .raw data was uploaded to Skyline for quantification. Normalized values were compared between samples and differences determined using a Wilcoxon matched-pairs signed rank test.

**SUPPLEMENTARY FIGURE CAPTIONS**

**Supplementary Figure S1: SDS-PAGE assessment of albumin and IgG depletion.** The Proteome blue depletion kit was used to **(A)** deplete the samples of **(B)** albumin and IgG to facilitate the (**C**) increased detection of low-abundant proteins from CRC patient plasma samples. SDS-PAGE was used to separate proteins based on mass and visualized with Sypro Ruby proteins stain.

**Supplementary Figure S2: Offline HILIC HPLC chromatograms of discovery proteomics used in label-free and quantitative (TMT) analysis.** Depleted plasma proteins from 9 CRC patient samples collected in **(A)** BCT or **(B)** EDTA tubes were pooled (1:1) and 20 g were fractionated by HILIC-HPLC. **(C-D)** Samples used in our TMT study were also fractionated into 12 HILIC fractions before being subjected to RP-LC-MS/MS, (Line indicates start of collection over 12 minutes, 1 per minute).

**Supplementary Figure S3: Quantitative assessment of CRC plasma proteins in paired tube collections, pre- vs. post-tumor resection.** Linear regression analysis of reporter-ion quantitation for proteins showing +/- 0.5 fold log2 change by TMT (184 total) in (**A**) Patient A (high-grade adenocarcinoma), and (**B**) Patient B (low-grade 561 adenocarcinoma). (**C**) Heat map-showing 8 proteins with +/- 0.5 fold change, pre- vs. post-operatively in both collection tube types via TMT analysis.

**SUPPLEMENTARY TABLES**

**Supplementary Table S1: CRC patients and healthy controls used to assess the stability of plasma proteins stored in BCT and EDTA by discovery and targeted proteomics.**

**Supplementary Table S2: (A) Listing of proteins identified by discovery proteomic in both BCT and EDTA tubes. (B) Listing of unique proteins identified by discovery proteomics in each collection tube.**

**Supplementary Table S3: Samples used for tandem mass tag (TMT) comparative and quantitative proteomic study.**

**Supplementary Table S4: Listing of proteins identify by comparative and quantitative analysis using tandem mass tags (A) experiment 1 and (B) experiment 2.**

**Supplementary Table S5: Listing of proteins showing +/- 0.5 fold change pre- vs. post operatively by comparative and quantitate proteomics.**

**Supplementary Table S6: PRM target inclusion list.**
